# Draft Genome Sequence of Stenotrophomonas daejeonensis Strain NLF4-10 Isolated from Livestock Wastewater

**DOI:** 10.1128/mra.00322-22

**Published:** 2022-08-08

**Authors:** Kook-Il Han, Young Ho Nam, Ji Young Jung, Sang Eun Jeong, Hyun Mi Jin, Mi-Hwa Lee

**Affiliations:** a Microbial Research Department, Nakdonggang National Institute of Biological Resources (NNIBR), Sangju-si, Gyeongsangbuk-do, Republic of Korea; Montana State University

## Abstract

Here, we report the draft genome sequence of Stenotrophomonas daejeonensis strain NLF4-10, isolated from a livestock wastewater treatment plant in Nonsan, Republic of Korea. The whole-genome sequence of S. daejeonensis strain NLF4-10 was analyzed using the Pacific Biosciences Sequel and Illumina NovaSeq sequencing platforms. The genome comprises a 3,655,148 bp chromosome with a GC content of 68%, 3,274 coding DNA sequences (CDSs), 59 transfer RNAs (tRNAs), and 4 noncoding RNAs (ncRNAs).

## ANNOUNCEMENT

Wastewater treatment plants emit various kinds of odor pollutants, like ammonia, hydrogen sulfide, volatile fatty acids, acetic acid, styrene, and other organic compounds (1–3). These malodorous gases in livestock manure adversely affect human beings, such as causing irritation to the nose, headache, dizziness, hyperemia, asphyxiation, nausea, and unconsciousness (4, 5). Recently, strain NLF4-10 was isolated during the investigation of the odor reducing bacteria.

The species S. daejeonensis MJ03^T^, which is Gram-stain-negative, motile, and rod shaped, was isolated from sewage treatment plants (6). Here, we describe the draft genome sequence and annotation of S. daejeonensis Strain NLF4-10 that was isolated from a livestock wastewater treatment plant in Nonsan, Republic of Korea. The biofilterfunctions as a livestock odor removal system. A liquid sample from the biofilter was immediately placed in a 4°C refrigerated room. The sample was 10-fold serially diluted in saline solution, and 100 μl of each dilution was spread on sulfur oxidation bacteria medium. The composition of sulfur oxidation bacteria medium was as follows: 8.0 g Na_2_S_2_O_3_·5H_2_O, 2.0 g K_2_HPO_4_, 2.0 g KH_2_PO_4_, 0.4 g NH_4_Cl, 0.2 g MgCl_2_·7H_2_O, 0.01 g FeSO_4_·7H_2_O, 15.0 g agar, 1L Distilled water at pH 7.0. After 5 to 10 days of incubation at 30°C, single colonies were selected.

The Strain NLF4-10 was grown in Trypticase soy agar (Difco, USA) for 5 days at 30°C. The genomic DNA was obtained from the cultivated cells on Trypticase soy agar (Difco, USA) using the Maxwell 16 DNA purification kit (Promega, USA). The extracted DNA was sequenced at Theragen Bio Institute (Republic of Korea) with a PacBio sequel using a 15 kb SMRTbell template library and Illumina Nova Seq platform (2 × 151-bp). A size selection cutoff of 7,000 bp was used. The size-selected SMRTbell library was annealed and bound according to the SMRT Link setup and sequenced on a Sequel II instrument. SMRTbell template prep kit 2.0 (PacBio) and TruSeq nano DNA library prep kit (Illumina) were used to prepare sequencing libraries. The 101,469 total reads (read N50, 11,224 bp) produced by the PacBio RS II platform were assembled, whereas the Illumina platform produced 15,357,866 total reads (read N50, 13,549 bp). Then, *de novo* genome assembly, error correction, and annotation were performed by Canu (v1.7) (7), Pilon (v1.22) (8), and Prokka (v1.1.0) (9) software, respectively. Aragon (v1.2.36) was used to predict tRNAs, and Barrnap software (v0.6) was used to predict rRNAs (5S rRNAs, 16S rRNAs, and 23S rRNAs) (10). To confirm species, the average nucleotide identity (ANI) was calculated using OrthoANI (11). Default parameters were used for all software unless otherwise specified.

The draft genome of strain NLF4-10 consisted of 4 contigs with a total length of 3,655,148 bp (read N50, 3, 578, 136). The sequencing depth of coverage was 236.0x and the genomic DNA GC content was 68.0 mol%. A total of 3,274 protein coding genes, 9 rRNA genes (3 of 5S rRNA, 3 of 16S rRNA, and 3 of 23S rRNA), 59 tRNA genes, 4 non-coding RNA, and 50 pseudo genes were predicted. Based on the 16S rRNA gene sequence similarity and average nucleotide identity, the strain NLF4-10 is most closely related to S. daejeonensis MJ03^T^ with values of 99.11% and 97.85%, respectively.

### Data availability.

The genome sequence and raw sequencing reads for strain NLF4-10 were deposited under GenBank accession numbers JAKSEE000000000, BioProject accession number PRJNA807444, BioSample accession number SAMN25981972, and Sequence Read Archive (SRA) accession number SRX15804104. The version described in this paper is the first version, JAKSEE010000000. S. daejeonensis strain NLF4-10 has been deposited in the Korean Collection for Type Cultures under accession number KCTC 14796BP.
